# Natural probiotics improve heritable sterility

**DOI:** 10.15252/embr.202358318

**Published:** 2023-11-20

**Authors:** Chee Kiang Ewe, Oded Rechavi

**Affiliations:** ^1^ Department of Neurobiology, Wise Faculty of Life Sciences and Sagol School of Neuroscience Tel Aviv University Tel Aviv Israel

**Keywords:** Evolution & Ecology

## Abstract

Disrupting the small RNA pathway and chromatin‐modifying enzymes in *C. elegans* often leads to a mortal germline (Mrt) phenotype, characterized by progressive sterility observed over multiple generations at elevated temperature. This phenotype arises from the inheritance of aberrant epigenetic memory across generations. In this issue of EMBO Reports, Frézal and colleagues reported that, while in standard laboratory environment *C. elegans* wild isolates exhibit the Mrt phenotype, sterility does not occur when the worms are exposed to naturally associated bacteria and microsporidia. Excitingly, diet‐induced epigenetic memory may persist for multiple generations. This suggests intriguing diet–gene interactions in modulating nongenetic inheritance, potentially shaping the evolutionary trajectory of the animals.

## Graphical abstract

The mortal germline phenotype, characterized by progressive sterility over multiple generations, is surprisingly prevalent in *C. elegans* wild isolates but suppressed by naturally associated bacteria and microsporidia.

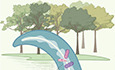

Since Sydney Brenner's introduction of *Caenorhabditis elegans* to the laboratory in 1970s, it has arguably become one of the best described animals on the planet and has been central to many major discoveries in biology, including systemic RNA interference (RNAi) in metazoan, and more recently, transgenerational epigenetic inheritance. Worms used in most laboratories are descendants of a single lineage, named N2, found in a mushroom compost in Bristol, gifted to Sydney Brenner by Ellsworth Dougherty. In the laboratory, *C. elegans* is cultured monoxenically on a uracil auxotroph *E. coli* strain called OP50—a B‐type strain—at 15–25°C.

Work in *C. elegans* over the past two decades has convincingly shown that epigenetic information can be transmitted across generations in animals. The heritable agents are small RNAs, which can be propagated by RNA‐dependent RNA polymerase (RdRP) and subsequently trigger chromatin modifications on the target loci via the nuclear RNAi pathway (Duempelmann *et al*, 2020; Ewe & Rechavi, 2023). Mutations in many genes functioning in the small RNA pathway (e.g., *prg‐1/PIWI* and nuclear argonaute *hrde‐1*) and chromatin modification (e.g., *spr‐5/LSD1* and *set‐2/SET1*) cause progressive sterility—a mortal germline (Mrt) phenotype—at high temperature (25°C), possibly owing to the accumulation of aberrant epigenetic memory across generations. It was proposed that inactivating RNAi machinery and chromatin modifiers causes downregulation of histone genes, defects in chromosomal pairing/segregation, and increased genome instability and transposon‐induced DNA damage, thereby compromising germ cell immortality (Katz *et al*, 2009; Buckley *et al*, 2012; Herbette *et al*, 2017; Barucci *et al*, 2020); however, the exact cause of sterility is debated.

While wild‐type N2 can be propagated at 25°C indefinitely, some wild isolates show Mrt phenotype at this temperature. Using linkage mapping, a previous study mapped the phenotypic variant to a deletion in *set‐24*, which encodes a presumptive lysine methyltransferase with a SET domain. It is noteworthy that the polymorphism in *set‐24* is very rare among *C. elegans* wild isotypes, possibly due to its detrimental effect on fertility (Frézal *et al*, 2018). In the new study from the same group, the authors expanded their findings to an impressive number (132) of wild isolates and found that more than half of the tested strains show the Mrt phenotype at 25°C. Using genome‐wide association (GWA) mapping, they found a major quantitative trait locus on chromosome III. The causal effect of chromosome III was further confirmed with near isogeneic lines (Frézal *et al*, 2023).

Considering that Mrt imposes a deleterious effect on fitness, the high prevalence of this trait in *C. elegans* natural population is puzzling. How are variants that cause Mrt maintained in the population? As *C. elegans* is fed bacterial monocultures in standard laboratory environments, the authors tested whether naturally associated microbes could affect the Mrt phenotype. Indeed, they found that worms freshly isolated with their associated microbial fauna can be propagated at 25°C for an extended period of time. However, once these worms were “bleached” (treated with alkaline hypochlorite) to remove the microbes, they started exhibiting the Mrt phenotype. Moreover, bacteria isolated from wild worms can at least partially suppress the Mrt phenotype. Interestingly, infecting the worms with a natural microsporidian pathogen can also rescue the Mrt phenotype (Fig 1). These results may be paralleled with a previous finding that activation of stress response limits epigenetic inheritance (Houri‐Zeevi *et al*, 2021). Of note, microsporidia are intestinal parasites and do not infect the *C. elegans* germline. Hence, their effects on germline immortality may reflect yet another example for soma‐to‐germline transfer of epigenetic information—breaching of the “Weismann Barrier”—and warrants future investigation.

**Figure 1:**
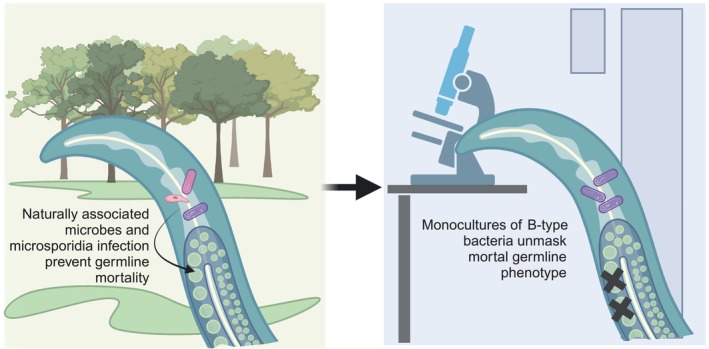
Environmental rescue of mortal germline (Mrt) phenotype in *C. elegans* wild isolates The Mrt phenotype in wild worms is rescued by their naturally associated microbial fauna and microsporidia. Standard cultivation with *E. coli* OP50 in the laboratory uncovers the Mrt phenotype in these worms, suggesting gene–diet interactions in modulating epigenetic reprogramming (created with BioRender.com).

Next, the authors compared the effects of different *E. coli* strains on the Mrt phenotype. They reported that K‐12, but not B‐type, strains can partially rescue the 25°C Mrt phenotype in wild isolates, as well as in epigenetic mutants (*nrde‐2*, *set‐2*, and *set‐24*) in the N2 background. Most excitingly, worms reared on K‐12 bacteria for several generations showed delayed onset of Mrt phenotype after returning to OP50, providing evidence for the *transgenerational* inheritance of diet‐induced epigenetic memory. Other previous studies have shown different dietary composition and starvation could trigger a heritable response in worms, and similarly, in mammals, parental diets have been shown to affect metabolic phenotypes of their progeny, but most of the reports concern relatively short‐lived *intergenerational* and not transgenerational effects (Guo *et al*, 2020).

While the molecular mechanisms underlying transgenerational epigenetic inheritance involving small RNAs are heavily studied, their impacts on ecology and the evolutionary trajectory of the animals remain less clear. Frézal *et al* showed that the Mrt phenotype in wild worms results from context‐dependent mutation(s), behaving neutrally in the wild but exhibiting deleterious effects in the controlled laboratory environment. Given the intimate connections between RNAi, innate immunity, and germline maintenance in *C. elegans*, it is tempting to speculate that the variants that produce Mrt phenotype are maintained by balancing selection and may function to fine‐tune the dynamics of epigenetic inheritance in the wild. This careful study provides valuable insights into how gene–environment interactions can impact epigenetic inheritance and opens many new questions: (i) What are the polymorphisms that produce the Mrt phenotype in wild isolates and how do they influence RNAi inheritance? (ii) How does the soma/intestine communicate with the germline to ensure germline immortality at elevated temperature? (iii) And finally, how do the bacteria affect epigenetic reprogramming in the germline? By leveraging the extensive polymorphism in wild worms and taking a closer look at their natural biotic environment, future studies promise to further unveil the roles of epigenetic inheritance in ecology and evolution.

## Disclosure and competing interests statement

The authors declare that they have no conflict of interest.
